# Validation of a continuous, arterial pressure-based cardiac output measurement: a multicenter, prospective clinical trial

**DOI:** 10.1186/cc6125

**Published:** 2007-09-19

**Authors:** William T McGee, Jeffrey L Horswell, Joachim Calderon, Gerard Janvier, Tom Van Severen, Greet Van den Berghe, Lori Kozikowski

**Affiliations:** 1 Critical Care Division, Baystate Medical Center, 759 Chestnut Street, Springfield, MA, 01199, USA; 2 Department of Cardiac Anesthesia, Medical City Dallas Hospital, 7777 Forest Lane, Dallas, TX, 75230, USA; 3 DAR II, CHU Bordeaux Group Hospitalier Sud, Avenue de Magellan, 33604 Pressac Cedex, France; 4 Department of Intensive Care Medicine, UZ Leuven Gasthuisberg, Catholic University of Leuven, B-3000 Leuven, Belgium

## Abstract

**Introduction:**

The present study compared measurements of cardiac output by an arterial pressure-based cardiac output (APCO) analysis method with measurement by intermittent thermodilution cardiac output (ICO) via pulmonary artery catheter in a clinical setting.

**Methods:**

The multicenter, prospective clinical investigation enrolled patients with a clinical indication for cardiac output monitoring requiring pulmonary artery and radial artery catheters at two hospitals in the United States, one hospital in France, and one hospital in Belgium. In 84 patients (69 surgical patients), the cardiac output was measured by analysis of the arterial pulse using APCO and was measured via pulmonary artery catheter by ICO; to establish a reference comparison, the cardiac output was measured by continuous cardiac output (CCO). Data were collected continuously by the APCO and CCO technologies, and at least every 4 hours by ICO. No clinical interventions were made as part of the study.

**Results:**

For APCO compared with ICO, the bias was 0.20 l/min, the precision was ± 1.28 l/min, and the limits of agreement were -2.36 l/m to 2.75 l/m. For CCO compared with ICO, the bias was 0.66 l/min, the precision was ± 1.05 l/min, and the limits of agreement were -1.43 l/m to 2.76 l/m. The ability of APCO and CCO to assess changes in cardiac output was compared with that of ICO. In 96% of comparisons, APCO tracked the change in cardiac output in the same direction as ICO. The magnitude of change was comparable 59% of the time. For CCO, 95% of comparisons were in the same direction, with 58% of those changes being of similar magnitude.

**Conclusion:**

In critically ill patients in the intensive care unit, continuous measurement of cardiac output using either APCO or CCO is comparable with ICO. Further study in more homogeneous populations may refine specific situations where APCO reliability is strongest.

## Introduction

Clinicians monitor hemodynamic variables to diagnose conditions and to follow treatment in critically ill patients. In the intensive care unit (ICU) and the operating room, such monitoring often includes cardiac output and, although potentially measured by newer techniques, usually requires placement of a pulmonary artery catheter. Intermittent (bolus) thermodilution cardiac output (ICO) measurement is a standard to which other methods of cardiac output measurement are compared [1]. Pulmonary artery catheterization has come under increasing criticism regarding its risks and costs, and questions have arisen about its benefits [2,3]. Technologies equally effective yet less invasive, safer, and simpler to use have consequently been sought for cardiac output monitoring [4,5]. One of the more promising approaches in the monitoring of cardiac output is the estimation of flow from analysis of the arterial pressure waveform.

Approaches to measuring cardiac output via a peripheral artery catheter typically use algorithms by which the pulse wave is analyzed and then related to a numerical value for cardiac output. These devices often require frequent calibration to initiate monitoring and to accurately assess cardiac output during changing of the vascular tone [6,7]. A new arterial pressure-based cardiac output (APCO) device uses access to the radial or femoral artery via a standard arterial catheter. This system (Vigileo/FloTrac; Edwards Lifesciences LLC, Irvine, CA, USA) allows determination of the stroke volume based on arterial waveform characteristics and individual patient demographics, without calibration [8-11].

This study compares measurement of cardiac output by analysis of the arterial pulse using APCO with measurement by ICO. The study was designed to determine whether cardiac output measurements obtained using APCO are comparable with those obtained using a clinically accepted method such as room-temperature ICO [12,13]. Continuous cardiac output (CCO) measured with a pulmonary artery catheter was also compared with ICO in order to show the performance of a widely used CCO measure against ICO. The less-invasive APCO technology may provide an additional option to improve hemodynamic management in critically ill patients, including those who currently are not monitored via pulmonary artery catheter but for whom continuous measurement of cardiac output and other flow-related parameters may allow timely identification of changes in hemodynamic status and rapid adjustment in therapy.

## Materials and methods

Adult patients requiring pulmonary catheters and radial or femoral artery catheters as part of standard clinical care were enrolled from 1 August to 15 December 2004, at two US sites and two European sites (Baystate Medical Center, Springfield, MA, USA; Medical City Dallas Hospital, Dallas, TX, USA; Centre Hospitalier Universitaire, Bordeaux Group Hospitalier Sud, Pessac, France; and Universitaire Ziekenhuizen Leuven, Leuven, Belgium). Each site enrolled at least 20 patients.

Pulmonary artery catheters (models 746HF8, 744HF75, 777HF8, or 774HF75; Edwards Lifesciences) were placed according to standard clinical practice for continuous and intermittent measurement of cardiac output using Vigilance™ monitors (Edwards Lifesciences). These catheters are equivalent in their ability to measure ICO and CCO. Catheter models differ in that some contain an additional volume infusion port, and some have the ability to measure right ventricular end-diastolic volume.

Radial and femoral arterial lines from a variety of manufacturers were connected to FloTrac™ sensors (Edwards Lifesciences), and the cardiac output was determined using the algorithm used in the commercially available Vigileo™ APCO system (Edwards Lifesciences) [8]. Hemodynamic data were monitored and recorded continuously and simultaneously with CCO and APCO, and intermittently using ICO. All hemodynamic data were collected on laptop computers and downloaded to a remote system for analysis.

For each patient, data collected from the APCO device were compared with simultaneously collected data from the pulmonary artery catheter over a 24-hour period. During the first 12 hours of data collection, reference ICO measurements were collected every 3 hours. During the second 12 hours, these measurements were made every 4 hours. All measurements were made in the ICU. The intervals for data collection were established to mimic the standard of care for cardiac output measurements of the participating institutions. ICO values were obtained from the average of a minimum four room-temperature saline boluses injected at various times during the respiratory cycle [14]: inspiration, peak inspiration, expiration, and end expiration. Additional ICO measurements depended on physician judgment and institutional practice. The physicians responsible for the care of these patients were usually the investigators. At least four complete sets of measurements were made for each patient. Cardiac output measurements derived from the APCO method were not used to guide therapy.

Baseline demographics and significant comorbidities were recorded in a database for subsequent analysis, and patient identifiers were removed.

Cardiac output data were collected from all patients. Data consisted of cardiac output determined by APCO, CCO, and ICO during reference measurements every 3 or 4 hours throughout the monitoring period. Bias and precision analysis were used to compare cardiac output measurements from the pulmonary artery catheter with those calculated from the APCO technology. Bland–Altman plots were generated [15]. The difference between APCO and ICO values and the difference between CCO and ICO values were determined for each set of cardiac output measurements. The mean and standard deviation of the difference between cardiac output measurements were calculated to estimate the bias and the precision.

The ability to accurately measure change in cardiac output is important in clinical practice [16]. Although a clinically relevant change in cardiac output is unknown, for the purposes of our analysis we defined a significant change in cardiac output as 30%. In analysis of the direction and the magnitude of change in cardiac output, the change in cardiac output (ΔCO) was calculated as the difference in cardiac output at two time points divided by the mean cardiac output at those two time points. ΔCO was expressed as a percentage by multiplying this quantity by 100%: ΔCO% = [CO_i _- CO_i-1_]/[(CO_i _+ CO_i-1_)/2] × 100%. Increases and decreases of the same magnitude had equivalent percentage changes that were opposite in sign.

The study protocol was approved by the institutional review boards and/or ethics committees of the participating sites. All patients or their legal guardians provided prior written informed consent for participation in this study.

## Results

Each of the study's four centers enrolled 20–23 patients, for a total of 86 enrolled patients. One patient died after only one dataset was collected, and another patient had no data logged due to technical difficulties. Of the remaining 84 patients, 69 had catheters placed during surgical procedures in the operating room before admission to the ICU. The other 15 participants were nonsurgical critical care patients. All data were obtained in the ICU. All patients had pulmonary artery catheters placed, and all but one patient also had a radial artery catheter inserted. One patient received a femoral artery catheter but no radial artery catheter, and another patient had radial and femoral artery catheters placed.

Approximately two-thirds of patients were male. Patients' ages ranged from 24–84 years, with a mean age of 68 years (Table 1). Patients had various comorbid diseases, and physicians placed pulmonary artery catheters for a variety of reasons (Table 2).

**Table 1:** Patient characteristics

	Males (*n *= 55)		Females (*n *= 29)	
	
	Mean	Range^a^	Mean	Range^a^
Age (years)	67	24–84	69	45–83
Height (cm)	174	160–185	160	148–172
Weight (kg)	88.2	60.0–150.7	69.3	41.2–112.7
Body surface area (m^2^)	2.07	1.66–2.54	1.71	1.33–2.11
Heart rate (beats per min)	86	57–116	87	57–117
Cardiac output (l/min)^b^	6.2	3.1–9.2	4.6	1.7–7.5
Cardiac index (l/min/m^2^)	3.01	1.74–4.29	2.7	1.38–3.96
Stroke volume (ml)	72.2	37.7–106.8	54.4	16.1–92.8
Mean arterial pressure (mmHg)	73.0	49.5–96.5	72.0	45.8–98.3

^a^For age, height, weight, and body surface area, ranges are minimum–maximum; for heart rate, cardiac output, cardiac index, stroke volume, and mean arterial pressure, ranges are ± 2 standard deviations. ^b^Mean cardiac output as measured by arterial pressure-based cardiac output.

**Table 2:** Most frequent patient comorbidities and most frequent reasons for pulmonary artery catheter insertion

Patient comorbidity	*n *(%)	Reason for pulmonary artery catheter insertion	*n *(%)
Systemic hypertension	48 (57)	Cardiac surgery	23 (27)
Coronary artery disease	29 (34)	Diagnosed cardiac disease	23 (27)
Valvular heart disease	28 (33)	Volume status	21 (25)
Diabetes	27 (32)	Perioperative monitoring	17 (20)
Hyperlipidemia	23 (27)	Multisystem organ failure	8 (10)
Angina	22 (26)	Acute heart failure	6 (7)
Arrhythmia	20 (24)	Severe sepsis	4 (5)
Congestive heart failure	18 (21)		

Multiple comorbidities coexist in many patients. In several patients, more than one reason was listed for pulmonary artery catheter insertion.

The bias of APCO compared with ICO was 0.20 l/min. The bias of CCO compared with ICO was 0.66 l/min.

**Figure 1:**
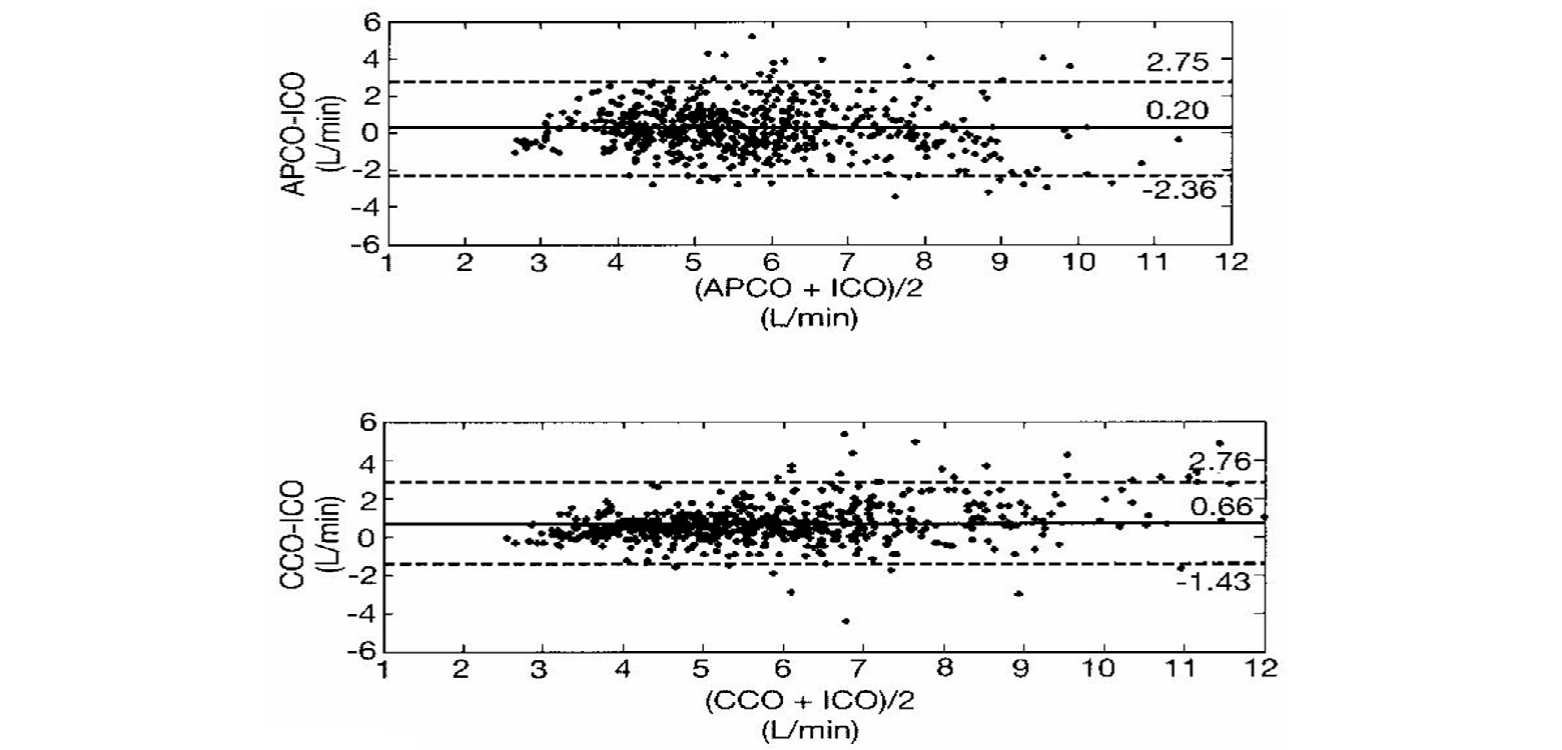
Mean difference in cardiac output as a function of mean cardiac output. Mean difference in cardiac output, measured by arterial pressure-based cardiac output (APCO) and intermittent thermodilution cardiac output (ICO) or measured by continuous cardiac output (CCO) and ICO, as a function of mean cardiac output. The difference in cardiac output as determined by the two methods is plotted against the mean cardiac output: upper, (APCO + ICO)/2; lower, (CCO + ICO)/2. Central solid line, mean difference; dashed lines, limits of agreement (95% confidence intervals). *n *= 84 patients; 561 data points.

For APCO relative to ICO, the precision was found to be ± 1.28 l/min. The precision for CCO relative to ICO was ± 1.05 l/min. The limits of agreement for APCO versus ICO were -2.36 to +2.75 l/min, and those for CCO versus ICO were -1.43 to +2.76 l/min. Figure 1 shows the distribution of the difference between cardiac output measured by APCO and ICO plotted against the mean cardiac output determined by the two methods [17]. The limits of agreement and the mean difference are shown. The figure also shows CCO versus ICO plotted in a similar fashion. The coefficient of variation for ICO was 18%.

**Figure 2:**
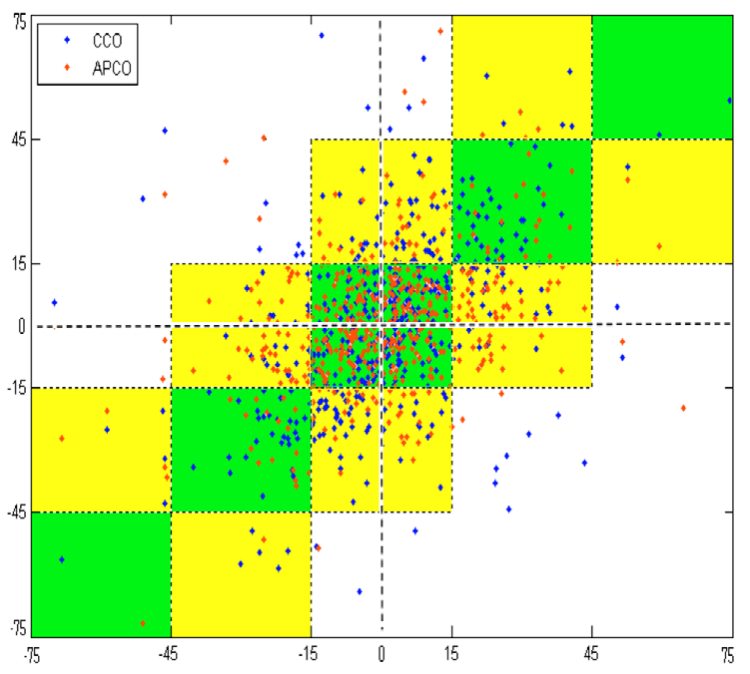
Change in cardiac output. The change in cardiac output (ΔCO) measured by intermittent thermodilution cardiac output (ICO) and by either arterial pressure-based cardiac output (APCO) or continuous cardiac output (CCO). ΔCO is the difference in two measurements (by one method) of cardiac output expressed as a percentage of the mean of those measurements. Points that fall within squares along the central diagonal (green squares) reflect equivalent changes for the test cardiac output measurement method (APCO or CCO) and ICO. Points that fall within the yellow squares reflect changes of similar direction but different magnitudes. Points that fall within white sections in the upper left and lower right reflect non-correlated changes between the test measurement method and ICO.

Changes in cardiac output are plotted in Figure 2. When ΔCO was measured by APCO, 59% of the time its magnitude and direction of change were within ± 15% of the ICO measurement (Figure 2; for example, ΔCO between -15% and +15% when measured by APCO, and ΔCO between -15% and +15% when measured by ICO). In 96% of ΔCO determinations, the APCO magnitude and direction of change were within ± 30% of the measurement of ICO (Figure 2; for example, ΔCO from -15% to +15% as measured by APCO, but from -45% to -15% or from +15% to +45% as measured by ICO). In 4% of the determinations of ΔCO, the APCO measurement direction and magnitude of change differed more than ± 30% from the measurements by ICO (Figure 2). For CCO compared with ICO, the respective percentages were 58%, 95%, and 5% for change within ± 15%, for change within ± 30%, and for change greater than ± 30%.

## Discussion

Our data demonstrate that APCO covaries with ICO in a series of critically ill patients over their initial 24 hours of ICU monitoring. The study population included patients with cardiac disease, multisystem organ failure, acute heart failure, and severe sepsis, as well as patients needing postoperative monitoring for cardiac surgery. Extensive data were gathered for 24 hours, comparable with studies of other methods for measuring cardiac output [18-20]. Considering the limitation of the differences in measurement techniques comparing a continuous measure that gives a running average of cardiac output over 20 seconds (APCO) versus ICO, which traditionally is obtained with a 4-second injection, the APCO performance was similar to the well accepted thermodilution CCO methodology that averages cardiac output over several minutes. Rapid dynamic changes in cardiac output that are seen in the clinical intensive care setting will contribute to the measurement differences observed in our patients. Averaging cardiac output over longer time periods with thermodilution CCO may not well represent the actual dynamic variation in stroke volume (SV) and cardiac output when measured against techniques that evaluate CO during shorter time intervals.

The present study is one of the largest clinical comparison studies of cardiac output monitoring [10,16,21]. We observed similar cardiac output measurements when comparing CCO with ICO, consistent with previous studies [18-20,22,23]; when compared with ICO, APCO measurements appeared to be less biased overall than CCO measurements.

The standard deviation of the difference between measurement by APCO (or CCO) and ICO gives an estimate of the precision of the APCO (or CCO) measurement compared with the ICO measurement [15]. When comparing two imperfect methods of measurement that each have an error distribution, the resulting error distribution (in this case) of the differences is wider than either of the two methods' error distributions, because overestimation by one method will occasionally be compared with underestimation by the other. For the measurement of cardiac output, ICO is the most widely accepted standard. ICO typically has an error (standard deviation) of 10–20% [13,18,21]; the ICO error was 18% in our patients. In our study, the overall 'grand mean' cardiac output over all patients by all three methods of measurement was 5.9 l/min. The observed standard deviation for the difference between APCO measurement and ICO measurement (± 1.28 l/min) was 1.28/5.9 = 22% of the grand mean cardiac output. The observed standard deviation for the difference between CCO and ICO (± 1.05 l/min) was 1.05/5.9 = 18% of the grand mean. The standard deviations for either method of continuous measurement of cardiac output observed in the present study are consistent and similar to the ICO error on serial measures we obtained under real ICU conditions.

Limits of agreement have been used in discussions about comparisons of measurement methods. If 15% is the typical precision of ICO [21], then the limits of precision (95% confidence limits) are ± 30% – an error considered clinically acceptable [18]. Two equivalent methods of measurement, each having ± 30% limits of precision, would have limits of agreement for their difference of ± 42%. The APCO versus ICO agreement of ± 43% (± 2 × standard deviation/mean cardiac output = ± 2 × 1.28/5.9) and the CCO versus ICO agreement of ± 36% (± 2 × standard deviation/mean cardiac output = ± 2 × 1.05/5.9) found in this study were therefore expected. Other investigators have suggested that two equivalent methods of measurement should have limits of agreement for differences of 28% [18]. That conservative estimate, however, assumed precision of 10% for the methods of measurement – greater precision than generally is accepted for thermodilution [13,18,21], and significantly better than the 18% observed in this study.

Clinical ΔCO values related to pathophysiology or treatments determine therapy at the bedside. Between method pairs (between APCO and ICO or between CCO and ICO), measurements of ΔCO by APCO compared with ICO were either of the same magnitude/in the same direction or were in the same direction/of lesser or greater magnitude within an overall ± 30% difference in magnitude in 96% of the paired measurements. More specifically, measurements were in the same direction and of the same magnitude as ICO (± 15%) in 59% of comparisons. They were dissimilar to ICO in 4% of comparisons. This compares favorably with CCO measurements of ΔCO, which were in the same direction and magnitude as ICO in 58% of comparisons, were in the same direction with ± 30% magnitude of change in 95% of comparisons, and were disparate to ICO in 5% of comparisons. This comparison of the magnitude and the direction of change avoids the problem of exaggeration of inaccuracies at high values when comparing absolute changes measured by two systems and at low values when comparing relative (percentage) changes.

There are significant limitations to our study. The variability in the reference measure of ICO is higher than generally accepted. When comparing the continuous measures of cardiac output with the reference standard, this variability could allow the APCO technology to appear similar in reliability to CCO when in fact it is not. Further data must be generated in the controlled setting of the operating room in paralyzed patients to clarify this issue. Assuring accurate timing of cardiac output determination to the respiratory cycle will improve the reliability of ICO.

In assessing a diverse group of patients with various levels of vascular tone related to pathophysiology, vasopressors, volume status, or other therapies, it remains unclear to what degree this may impact the determination of cardiac output from a peripheral artery. Including patients with various degrees of vascular tone impacted by their clinical condition (that is, sepsis, multiorgan failure, and vasopressors) may limit the reliability of a technique that depends on arterial waveform analysis. Independent study of more homogeneous groups such as severe sepsis with or without vasopressors will be required to answer these important questions.

There are many examples of patient subgroups included in our population that require independent validation. Patient-specific issues related to vascular compliance and tone are the most obvious, but specific physiology, medications, and volume status may also impact on cardiac output measurement from analysis of the arterial pulse. Simply, cardiac output performance in the major shock categories warrants further investigation. The dynamic heterogeneity of our patients may limit evaluation of cardiac output utilizing the arterial pulse via a peripheral artery when compared with thermodilution. Studies in homogeneous populations under similar conditions may shed light on this issue. Other issues that would limit the utility of arterial pressure and waveform assessment related to the arterial pulse are limitations of the device. A high-fidelity reliable arterial waveform is essential to cardiac output determined in this manner. Significant aortic valvular disease or the presence of an intraaortic balloon pump would also be expected to influence cardiac output using arterial waveform analysis.

## Conclusion

In our patients, APCO showed acceptable bias, precision, and measurement of cardiac output compared with ICO (the current standard). Thermodilution CCO, utilizing a pulmonary artery catheter, showed similar bias and precision to continuous APCO when compared with ICO. APCO appears to be a promising minimally invasive method of CCO measurement that requires further investigation.

## Key messages

• APCO is a less invasive technique requiring simply an arterial catheter and does not require calibration or central venous access.

• APCO compares favorably with CCO methodology using a pulmonary artery catheter when bolus intermittent thermodilution is used as a reference in the ICU.

## Abbreviations

APCO = arterial pressure-based cardiac output; CCO = continuous cardiac output; ΔCO = change in cardiac output; ICO = intermittent thermodilution cardiac output; ICU = intensive care unit.

## Competing interests

Edwards Lifesciences (Irvine, CA, USA) provided a research grant for execution of the protocol described in Materials and methods. WTM and JLH have received consulting fees from Edwards Lifesciences. WTM is also on a speakers' panel for Edwards Lifesciences. All data were collected at the four clinical sites by the investigators. Edwards Lifesciences received the electronic data for their critique of the technical aspects of the data collection and analysis.

## Authors' contributions

WTM, JLH, GJ, and GVdB were responsible for study design, data interpretation, and drafting the manuscript. WTM, JLH, JC, TVS and LK were responsible for data acquisition and analysis.
